# Surgical Tuberculosis1Read by invitation in a symposium on tuberculosis at the midsummer quarterly meeting of the Medical Society of the County of Cattaraugus, August 5, 1902.

**Published:** 1902-11

**Authors:** G. E. Benninghoff

**Affiliations:** Bradford, Pa.


### Surgical Tuberculosis.[1]

By G. E. BENNINGHOFF, M.D., Bradford, Pa.

THE surgical part of this subject which has been assigned to me is supposed to deal with diseased conditions more amenable to surgical than medical treatment, or to those cases where it is best to combine both surgical and medical treatment, and the latter is by far the largest class. The surgeon, in thinking of the cases which should be surgical, is not blamable if he brings in his clinical experience, and claims the greater proportion of all, for there are but few cases of tuberculosis that, at some time during the course of the disease, do not have a surgical side. There is no form of the disease existing, wherever it may be, within any of the great cavities of the body or in parts external, that at some time does not present a surgical aspect. We may not always be able to definitely diagnosticate the disease and know that it is surgical, but usually it is present; and, again, what is surgical tuberculosis today perhaps was not so twenty-five, or ten, or even five years ago.

One may say that tuberculosis of the lung is most strictly a medical disease, but how often is the condition in this organ one for surgical intervention and one, too, in which, unless relieved by surgery, there is no relief to be found? But tuberculosis of the osseous system and joints is the pathological condition that must depend most and more often on surgery for cure, and it is here that surgery demonstrates most fully its power to overcome the disease. One may safely say that the success of surgery in bone and joint tuberculosis has been so instructive that it has greatly advanced the treatment of the disease elsewhere in the body. Tuberculous peritonitis would have remained an exclusive medical disease had not surgery of the joints demonstrated its cure by surgery. At the present time, if taken early, the surgeon can promise quite as much in tuberculous disease of the peritoneal or pelvic cavities as he can in disease of the joints due to the same cause.

Tuberculosis is a disease which can exist in almost any part of the body, and it is one too often not recognised soon enough to give either the patient or the surgeon the great benefit of an early cure by operative treatment. It is not infrequently that the surgeon sees patients who have been treated for months and

1. Read by invitation in a symposium on tuberculosis at the midsummer quarterly meeting of the Medical Society of the County of Cattaraugus, August 5, 1902.

even years, for rheumatism of a joint or bone, when the case is one of tuberculosis, and months before should have had operative treatment for the cure of the disease. A mistaken diagnosis between tuberculosis of the joints, where more than one part is affected and rheumatism is very easily made, and I have found myself at fault several times, even when suspecting the condition.

Another form of tuberculosis, which is often mistaken for something else, is tuberculous pelvic disease. This condition is most deceptive, because it frequently is wanting in all symptoms of tuberculosis. One expects tuberculous disease to be accompanied by some thermic range. There should be some fever, and, again, the temperature should be subnormal and there should be loss of weight, some exhaustion and some pain, but very frequently not one of these symptoms will be present in pelvic tuberculosis. The physician's attention will be directed to the locality because of some menstrual or intestinal irregularity, or, possibly, sciatica or bladder symptoms. Examination reveals a mass in one side of the pelvic cavity or perhaps, one in either side, not painful on pressure, very hard, and generally not fixed, but quite frequently movable. For myself, I have usually taken this condition for some form of a tumor, until operation revealed its identity. In the beginning of my surgical work, I thought this kind of abscess nontuberculous, but during the last few years, I have usually been able to classify it, which has been verified by the microscope in a number of cases.

Another condition coming to the notice of the surgeon after considerable valuable time has been lost, is tuberculous empyema. A patient in whom tuberculosis has not been suspected has gone through with pneumonia, or a pleuropneumonia, and while the original disease has subsided, the patient does not make a good recovery. Usually the case is treated expectantly for several weeks, during which time the patient continually grows worse. About this time one begins to think of the possibility of pus in the pleural cavity, but examination gives little information. One looks for a pleural cavity full of pus, whereas in tuberculous cases it is usually encysted and may not at the time it should be discovered, contain more than two or three ounces of pus. Later, when the golden moment has passed, one can discover it with ease.

Tuberculous adenitis is another form of the disease which the surgeon very often finds neglected. The enlarged glands, especially when they occur in the groin, or inguinal region, are often considered syphilitic, or when they follow scarlatina and diphtheria, and are located in the cervical region, they are thought to be due to one or the other of those diseases. Wherever they may become tuberculous, they are very often thought to be due to some other condition than tuberculosis. The importance of recognising this condition as tuberculous, and immediately extirpating the diseased glands must be apparent to all, for when a tuberculous gland is allowed to remain in the body until it breaks down into caseous liquefaction, the danger of spreading the disease through the entire system is well known, and the surgeon usually sees these cases after this has occurred. As further examples, tuberculous kidney, skin and testicle, might be given as among those frequently overlooked. Time will not permit me to detail the numerous other tuberculous conditions which should be, but often are not diagnosticated early enough.

The successful treatment of surgical tuberculosis depends greatly upon an early diagnosis. The degree of disease present in the part affected is in direct ratio with the number of cures. This is well demonstated by the fact that in tuberculous joints, if the disease is taken early, it may be cured by medico-surgical treatment, or by erasion instead of excision, and the percentage of cures is in favor of either of these methods. When tuberculous disease of any joint, except possibly the hip, has advanced so far that excision is necessary, amputation of the limb above the joint affected is probably the operation best suited to the case, for thus one is sure of having removed the disease with little pain, little time wasted in bed, and little exhaustion. Thus an early diagnosis means early operative treatment, which usually saves the limb, while a late diagnosis means amputation, or its equivalent.

Not only is an early diagnosis necessary for a surgical cure, but also for any other method, whether medical or medico-surgical. At the present time, surgeons are becoming more conservative in the heroic treatment of tuberculous joints, while in treating the disease where it is purely surgical, as that of the pelvic or peritoneal cavity, kidneys, or glandular system, early and heroic methods are used. Inasmuch as ankylosis of a tuberculous joint is the most perfect cure—except in that very limited number where medico-surgical methods cure and preserve the physiological functions of the joint—any treatment that may cure, either by ankylosing the joint, or that will cure and allow the functions of the joint to remain, would seem the best. That being true, it follows that after becoming assured—and with the aid of the microscope there never need be much doubt—that the disease is tuberculous, one is justified in using any method that cannot produce a condition worse than an ankylosis.

To illustrate the proposition the elbow-joint may be taken as a hypothesis. The joint is found swollen, somewhat flexed, the articular ends of bones enlarged, more or less fluctuation present, motion and pressure painful. With this array of symptoms, and the previous history of the case, one is justified in presuming it tuberculous inflammation of the joint. Under strict antiseptic precautions, the joint can be aspirated and the fluid examined microscopically, thus verifying or nullifying the tuberculous idea of the disease. If there is conclusive evidence that it is tuberculous, one can do no harm by continuing the aspiration until all fluid possible has been drawn away, after which any substance desired may be introduced through the aspirating needle. After using Pennsylvania crude petroleum for two years in various ways as a local antiseptic and an antitubercular remedy, I do not hesitate to recommend it for use in this condition, and have done so in a few instances. I inject the joint with from one-half to two ounces of it. After it is introduced within the cavity of the joint, the parts should be kneaded, so as to distribute the fluid throughout the diseased parts of the structure.

Crude petroleum is very slowly absorbed by the tissues surrounding the parts where it is used. I have removed portions of bone which were tuberculous into the medullary canal, filled the cavity and the entire wound with petroleum, sutured the parts and observed it heal by immediate union, the bone in time becoming healthy. After introducing petroleum into the cavity of a joint, one should expect for two or three days an active reaction. The joint becomes swollen, the temperature of the body is increased from two to three degrees, and there is considerable pain, all of which subsides in the course of a week. Permit me further to state that my knowledge, gleaned from actual experience in the use of petroleum in tuberculous joints is yet limited, having used it in only three cases. Two of these recovered, one with function, the other with ankylosis. I am at present using it in another case, the joint under treatment being one of four different joints affected in the same patient, the elbow having been erased for tuberculosis. But I have used it in every form of septic wounds and other tuberculous diseases, than of osseous system, and in various ways, such as injecting it into the tissues, by syringing out cavities with it and by packing with gauze saturated with it, and I have seen the most perfect results follow its use.

I should be pleased if some of my professional friends here present would put the agent to the test in order to determine more fully as to its efficacy. In my surgical technique, all the assistants' hands are bathed in crude petroleum, after a thorough scrubbing in green soap and sterilised water. The oil is washed from the hands in alcohol. The oil completely closes all openings in the skin, and all germs are kept from coming to the surface of the skin of the operator's hands. A well-oiled hand is practically a gloved one, so far as preventing skin germs on the hands of the operator from infecting the field of operation.

Apologising for this digression, I wish to say further, that besides petroleum, one need not fear using any other substance in treating joint tuberculosis. The bichloride of mercury, iodoform, and the like, all or any kind of treatment may be used aiming to ankylosis as a cure. Failing to accomplish a cure in a reasonable time, the joint should be erased and ankylosis produced. Failing again, excision or amputation, one or the other, is to be considered.

